# Transient receptor potential melastatin 2 ion channel activity in ovarian hyperstimulation syndrome physiopathology

**DOI:** 10.3906/sag-2005-297

**Published:** 2021-04-30

**Authors:** Cengiz ŞANLI, Remzi ATILGAN, Tuncay KULOĞLU, Şehmus PALA, Bilge AYDIN TÜRK, Hasan Burak KESER, Nevin İLHAN

**Affiliations:** 1 Department of Obstetrics and Gynecology, Faculty of Medicine, Fırat University, Elazığ, Turkey; 2 Department of Histology and Embryology, Faculty of Medicine, Fırat University, Elazığ Turkey; 3 Department of Pathology, Faculty of Medicine, Adıyaman University, Adıyaman Turkey; 4 Department of Biochemistry, Faculty of Medicine, Fırat University, Elazığ Turkey

**Keywords:** Rat, OHSS, TRPM2, VEGF, angiogenesis

## Abstract

**Background/aim:**

Ovarian hyperstimulation syndrome (OHSS) is a complication of ovarian stimulation with increased vascular endothelial growth factor (VEGF) and vascular permeability in the ovarian tissue. Transient receptor potential melastatin 2 (TRPM2) is known to be associated with angiogenesis and vascular permeability. In this experimental study, we aimed to investigate the activity of TRPM2 in the development of OHSS.

**Materials and methods:**

Fourteen immature female rats were divided into two groups. Group 1 was the control group, and Group 2 was the OHSS group that was exposed to 10 IU of subcutaneous application of FSH for four days and 30 IU of human chorionic gonadotropin (hCG) on the 5th day. At the end of the experiment, the ovaries were removed. The right ovarian tissues were stored in 10% formol for histopathological and immunohistochemical examination. The left ovarian tissues were stored at –80 °C for biochemical examinations. VEGF, tumor necrosis factor-alpha (TNF‐α) and malondialdehyde (MDA) levels were measured in the ovarian tissue. Congestion, edema, apoptosis and TRPM2 immunoreactivity were evaluated.

**Results:**

There was a significant increase in ovarian weight in the OHSS group compared to the control group. There was a significant increase in congestion, edema, apoptosis and TRPM2 immunoreactivity in the OHSS group. A significant increase in tissue levels of VEGF, TNF‐α and MDA was also found in the OHSS group compared to the control group.

**Conclusion:**

As a result of our experiment, it was found that increased TRPM2 immunoreactivity on hyperstimulated rat ovary may be the reason or result of edema and congestion. Further studies are needed to discuss our results.

## 1. Introduction 

Ovarian hyperstimulation syndrome (OHSS) is the most serious complication associated with assisted reproductive treatments. Its pathophysiology includes increased capillary permeability which leads to a variety of signs and symptoms, such as hypotension, ascites and pleural effusion [1]. While mild OHSS is seen in 32% of in vitro fertilization (IVF) cycles, 10%–15% of patients develop moderate and 5%–8% severe OHSS [2,3]. Generally, it is believed that human chorionic gonadotropin (hCG) hormone is the most important factor for the development of this life-threatening condition [4]. The increased secretion of some mediators such as interleukins (ILs), vascular endothelial growth factor (VEGF), which occupies a special place for interleukins, angiotensin II (Ag II), insulin-like growth factor‐1 (IGF‐1), epidermal growth factor (EGF), transforming growth factor (TGF) a and b, basic fibroblast growth factor (bFGF), platelet-derived growth (PDGF) by granulosa and lutein cells lead to increased capillary permeability and accumulation of fluid in the third space [5]. Recent studies have shown that these molecules are responsible for the inflammatory processes associated with follicular maturation, ovulation, corpus luteum function and embryo implantation [5]. It has been reported that other mediators such as histamine, prolactin, prostaglandins and renin‐angiotensin also play a role in the pathophysiology of OHSS. As OHSS progresses, it leads to ascites formation and⁄or pleural effusion, loss of fluid from the vascular compartment (hemoconcentration), decreased kidney perfusion, followed by oliguria, kidney failure, and thrombosis [6]. 

Transient receptor potential melastatin 2 (TRPM2) channels are voltage independent cation channels which have been detected in tissues such as brain, bone marrow, lung, heart, pancreatic beta cells, leukocytes and vascular endothelium, and they are permeable to calcium, an important second messenger [7,8]. There are three extracellular factors which are effective on the opening of active TRPM2 cation channels: oxidative stress, ADPR/nicotinamide adenine dinucleotide (NAD+) metabolism and tumor necrosis factor-alpha (TNF‐α) [9]. Malondialdehyde (MDA), produced as a result of lipid peroxidation of cell membranes, is a metabolite that reflects the imbalance between oxidative and antioxidative homeostasis [10]. In our study, we aimed to investigate the tissue TNF‐α and MDA levels together with TRPM2 activity due to their relationship with oxidative stress. 

Endothelial Ca2+ signals play a very important role in angiogenesis and arteriogenesis [11]. Multiple proangiogenic factors, such as VEGF, bFGF, PDGF, epidermal growth factor (EGF), stromal-derived factor-1α (SDF-1α) and angiopoietin, regulate endothelial cell behavior by causing an increase in intracellular Ca2+ concentration [12,13]. TRP channels regulate endothelial cell function by mediating extracellular Ca2+ input in response to chemical, mechanical, and thermal stimuli [14].

Endothelial TRP channels represent potential therapeutic targets in many disorders characterized by abnormal vascularization such as cancer, ischemic disorders, retinal degeneration, and neurodegeneration [15]. Due to the proven TRPM2 expression in granulosa cells of in situ human follicles [16] and its close association with angiogenesis, we aimed to investigate TRPM2 ion channel activity in OHSS pathophysiology in an OHSS rat model.

## 2. Materials and methods

### 2.1. Animal model

This experimental study was performed in the Fırat University Experimental Research Center (FÜDAM) between April 2019 and July 2019, after approval by the Local Ethics Committee of Fırat University Animal Experiments (Date: 16. 01. 2019, Meeting Number: 2019⁄01, Decision No: 14). All rats for the study were purchased from FÜDAM. All research animals were treated in compliance with the guidelines for the care and use of animals approved by the institution in accordance to principles of laboratory animal care (NIH Guide for the Care and Use of Laboratory Animals, Institute of Laboratory Animal Researches, National Research Council, Washington, DC, USA). The rats were caged in a controlled environment of 22 °C with 12 h light ⁄ dark cycles (lights‐on 8 AM to 8 PM). Standard rat feed and reverse-osmosis-purified water were provided ad libitum. All the researchers participating in the experimental study had a certificate of animal use in experimental research.

### 2.2. Experimental protocol

We used immature rats (22 days old) in our experiment because ovarian physiology could be simplified and immature rats were not affected by the corpus luteum of previous cycles [17]. Twenty-two‐day‐old female Wistar albino rats (each weighing between 50 and 60 g) were randomly divided into two groups.

Group 1 (G1) (control group; n = 7): the rats received 0.1 mL subcutaneous saline from days 22–26. These rats were decapitated on 27th day.

Group 2 (G2) (OHSS group; n = 7): the rats were given with 10 IU of pregnant mare serum gonadotropin [PMSG; Folligon, Intervet India Pvt. Ltd (MAH-India), Pune, India] subcutaneous for 4 consecutive days from the 22nd to the 25th day of life followed by a subcutaneous injection of 30 IU of hCG [PREGNYL 1500 IU Lyophilized ampoule (Merck Sharp Dohme İlaçları Ltd. Şti., İstanbul, Turkey) contains 1500 IU (international units) of hCG in each ampoule] on the 26th day to induce OHSS. The rats were decapitated on the day after OHSS induction (27th day). 

The rats were monitored daily, and no evidence of toxicity was reported based on body weight, food consumption, grooming behavior or activity levels compared to controls. 

The rats were anesthetized with an intramuscular injection of ketamine (50 mg/kg; Ketalar; Parke Davis, Turkey) and xylazine (10 mg/kg; Rompun; Bayer AG, Berlin, Germany) under aseptic conditions. The abdomen was opened with a midline incision. On the 27th day, in G1 and G2, rats’ abdomens were exposed under anesthesia to see whether there was any ascites, and the ovaries were removed and weighed. Then, left ovarian tissues were wrapped in aluminum foil and stored at –80 oC. The right ovarian tissues were kept in 10% formol. At the end of experiment, all rats were sacrificed. The all samples were stored until the laboratory examinations were carried out under appropriate conditions.

### 2.3. Biochemical evaluation

All of the biochemical evaluations were done in Fırat University Medical Faculty, Medical Biochemistry Laboratory.

#### 2.3.1. Preparation of tissue samples

Fresh ovarian tissues were washed with 0.9% cold ( +4 ºC) sodium chloride (NaCl solution, 9 g/L) and dried with blotter paper. The tissues were then homogenized with a homogenizer (Ultra Turrax Type T25‐B, IKA-Werke GmbH, Staufen, Germany) in 0.01M phosphate buffered saline (PBS) solution [(10 mM Na2HP04, 10 mM KH2PO4, 0.9 g NaCl /100 ml and pH 7.4), Phosphate Buffered Saline, P4417, Sigma-Aldrich, St. Louis, MO, USA] for 3 min at 16000 rpm. Homogenization was carried out in an ice bucket. The homogenate was centrifuged at 5000 × g for 1 h (at + 4 ºC) and its supernatants were separated. The resulting supernatants were portioned to avoid repeated freeze-thaw cycles and stored at –80 °C to be thawed on the day of assay VEGF, TNF‐α, and malondialdehyde (MDA) levels were determined in the supernatant by enzyme-linked immunosorbent assay (ELISA) method. The results were normalized to the protein content in a sample and the estimation of protein content was performed by the Lowry method [18]. 

#### 2.3.2. Measurement of tissue VEGF Levels

VEGF levels in ovarian supernatant were determined by rat ELISA kit (SunRed Biotechnology Company, Shanghai, China, Catalog #: 201111636) according to manufacturer’s recommendations. The optical density of each well was measured at 450 nm using an ELX 800 ELISA reader. The concentrations were calculated based on standard curves. In plate washing, Bio‐tek ELX50 (BioTek Instruments, Winooski, VT, USA) was used as an automatic washer. Results were expressed in ng/mL. Tissue VEGF results were calculated by multiplying the dilution factor by considering dilution rate and expressed in ng/mg protein. The measurement range was 0.15–40 ng/mL and the minimum measurable level was 0.133 ng/mL.

#### 2.3.3. Measurement of tissue TNF‐α Levels

TNF‐α levels were measured in the tissue samples according to the kit procedure, using the rat ELISA kit (SunRed Biotechnology Company, Shanghai, China, Catalog #: 201110765). Absorbances were read spectrophotometrically at 450 nm in the ELX800 ELISA reader. In plate washing, Bio‐tek ELX50 (BioTek Instruments) was used as an automatic washer. Results are expressed in ng/L. TNF‐α results were calculated by multiplying by dilution factor considering the dilution rate. The tissue TNF‐α levels were expressed as ng/g protein. The measuring range was 8–1000 ng/L and the minimum measurable level was 5.127 ng/L. 

#### 2.3.4. Measurement of tissue MDA Levels 

MDA levels were measured using rat MDA ELISA kit (SunRed Biotechnology Company, Catalog #: 201110157) according to the manufacturer’s protocol.  The absorbance value (OD) of each pore was measured at 450 nm in the ELX800 ELISA reader. The results were normalized to the protein content in a sample and expressed in nmol/mg protein.

### 2.4. Histological evaluations

The all histological and immunohistochemical evaluations were done in Fırat University Medical Faculty Histology and Embryology Laboratory. The ovarian tissues obtained in each group were embedded in paraffin blocks after fixing with 10% formol. Sections of 4–6 µm thickness were obtained from those paraffin blocks. The sections were stained with hematoxylin and eosin (H&E), and examined and captured under the light microscope. The congestion and edema in ovarian tissue was scored on a scale ranging from 0 to 3 (0 = none; 1 = mild; 2 = moderate; 3 = severe) [19].

### 2.5. Terminal deoxynucleotidyl transferase dUTP nick end labelling (TUNEL) staining

Sections of 5–6 μm thickness obtained from paraffin blocks were mounted on polylysine glass slides. Apoptotic cells were detected using the ApopTag Plus Peroxidase In Situ Apoptosis Detection Kit (Chemicon International, Temecula, CA, USA, Cat no: S7101) according to the manufacturer’s instructions. Tissues deparaffinized with xylene were passed through graded alcohol series and washed with PBS. Tissues incubated for 10 min with 0.05% proteinase K were incubated for 5 min with 3% hydrogen peroxide to prevent endogenous peroxidase activity. After washing the tissues with PBS, they were incubated with equilibration buffer for 6 minutes and incubated for 60 min with a working solution (70% μL reaction buffer + 30% TdT enzyme) at 37 ºC. Apoptotic cells were visualized with the diaminobenzidine (DAB) substrate. The cross-staining sections with Harris hematoxylin were sealed with the appropriate capping solution. The prepared preparations were examined, photographed, and examined under computer‐assisted Leica DM500 light microscope (Leica Microsystems, Wetzlar, Germany). In the evaluation of TUNEL staining, blue‐stained nuclei by Harris hematoxylin were evaluated as normal, whereas cells displaying brown-stained nuclei were considered as apoptotic. At least 500 cells were counted in normal and apoptotic areas in randomly selected areas at × 10 magnification in sections. Apoptotic index (AI) was calculated as a percentage (%) by proportioning apoptotic cells to total (normal + apoptotic) cells [20].

### 2.6 Immunohistochemical staining experiments

Sections of 4–6 µm thickness were obtained from paraffin blocks and taken on polysynic slides. The deparaffinized tissues were passed through graded alcohol series and boiled in a citrate buffer solution for antigen retrieval at a pH of 6 in a microwave oven (750W) for 12 min. Tissues were kept at room temperature for about 20 min after boiling to cool. The cooled tissues were washed with PBS for 3 × 5 min, then incubated with hydrogen peroxide block solution (Hydrogen Peroxide Block, TA‐125‐HP, Lab Vision Corporation, Fremont, CA, USA) for 5 min to prevent endogenous peroxidase activity. Tissues were washed for 3 × 5 min with PBS applied Ultra V Block (TA‐125‐UB, Lab Vision Corporation) solution to prevent ground staining. After this procedure, the tissues were incubated with TRPM2 primary antibodies (Rabbit Anti‐TRPM2 antibody, ab101738, Abcam, Cambridge, UK) diluted at the rate of 1/200, at room temperature for 60 min. The tissues were washed with PBS for 3 × 5 min after primary antibody administration, and then incubated at room temperature in the humid environment for 30 min with the secondary antibody (biotinylated goat antipolyvalent (antimouse/rabbit IgG), TP – 125‐BN, Lab Vision Corporation). The tissues were washed for 3 × 5 min with PBS after secondary antibody administration. Then, after incubating with streptavidin peroxidase (TS ‐ 125‐HR, Lab Vision Corporation) for 30 min at room temperature in humid environment, it was taken into PBS. The 3‐amino‐9‐ethylcarbazole (AEC) Substrate + AEC Chromogen (AEC Substrate, TA‐015 and HAS, AEC Chromogen, TA‐002‐HAC, Lab Vision Corporation) solution was dropped onto the tissues. Following this procedure, after the image signal was received under a light microscope, the tissues were washed simultaneously with PBS. Tissues that were contrasted with Mayer’s hematoxylin were passed through PBS and distilled water and closed with the appropriate closure solution (Large Volume Vision Mount, TA‐125‐UG, Lab Vision Corporation). The preparations were examined, and photographed with a computer‐assisted Leica DM500 light microscope (Leica Microsystems GmbH, Wetzlar, Germany).

Histoscore was calculated for immunohistochemical staining. Histoscore = distribution (0.1 = under 25%; 0.4 = 26%–50%; 0.6 = 50%–75%; 0.9 = 76%–100%) × intensity (0 = no staining; + 0.5 = very little staining; + 1 = little staining; + 2 = medium staining; + 3 = very strong staining) [21].

### 2.7. Statistical analysis

When a power analysis of 80% power and 0.05 significance level was performed for the variable with the widest standard deviation from the variables to be used in the study, it was calculated that there should be at least 5 optimally 7 subjects in each group. SPSS 21.0 software (IBM Corp., Armonk, NY, USA) was used for the statistical analysis of data. The Kolmogorov–Smirnov and Shapiro–Wilk tests were used as tests of normality for the continuous variables. Nonnormally distributed data was expressed as the median (minimum‐maximum). Binary comparisons were done with Mann–Whitney U test. Categorical variables were compared with chi-square test and Fisher’s exact test variables? where applicable. A P value smaller than 0.05 was considered as statistically significant.

## 3. Results

### 3.1. Ovarian weights

**Figure 1:**
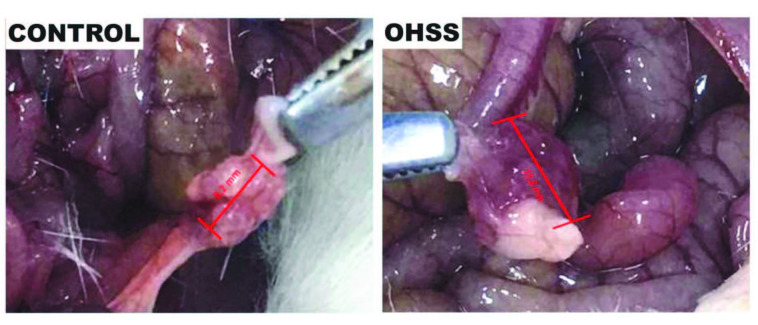
In the ovary with OHSS, increased ovarian size is observed compared to the ovary in the control group.

The ratio of ovarian tissue to body weight was significantly increased in OHSS group compared to the control group (P < 0.001), (Figure 1), (Table 1).

**Table 1:** The ratio of ovarian tissue to body weight.GroupsOvary / body weight (×10–3)G10.45 (0.28–0.59)G21.15 (0.89–1.66)aP value0.001Note: values are presented as median (min-max).G1 = Sham group; G2 = OHSS group.aCompared to control group

Groups	Ovary / body weight (×10–3)
G1	0.45 (0.28–0.59)
G2	1.15 (0.89–1.66)a
P value	0.001

### 3.2. Congestion and edema

**Figure 2:**
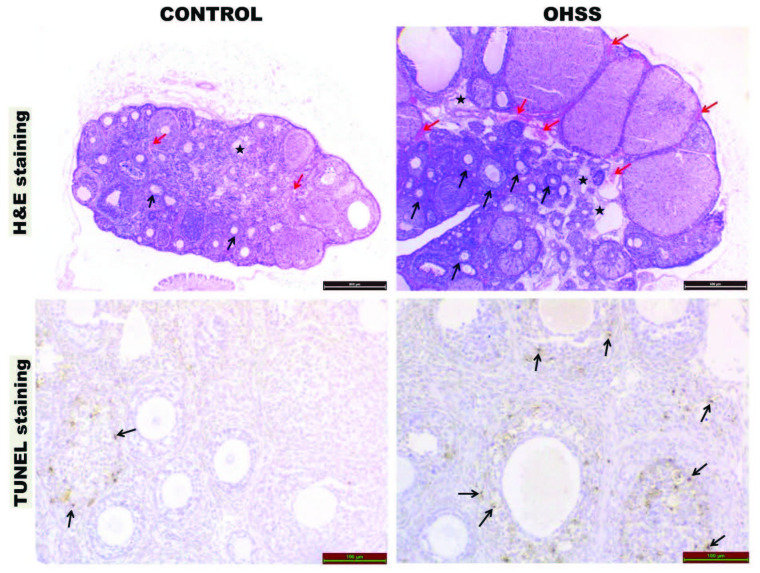
Hematoksilen & Eozin staining: significant ovarian size increase is observed in the OHSS group ( 4 magnification, scale bar = 500 μm) black arrow = follicles of various types; red arrow = congestion; black star = edema. TUNEL staining: TUNEL positive cells are observed in the granulosa cells of the follicles and in the stromal cells (black arrow).

There was a significant increase in congestion (P = 0.002) and edema (P = 0.001) in the OHSS group (Figure 2), (Table 2).

**Table 2:** Congestion, edema, TRPM2 immunoreactivity histoscore and apoptotic index (values are median (min-max).

Groups	Congestion	Edema	TRPM2	Apoptotic index (%)
G1	1.00 (0.00–1.00)	0.00 (0.00–1.00)	0.20 (0.10–0.30)	3.14 (2.00–5.00)
G2	2.00 (1.00–3.00)a	2.00 (1.00–3.00)a	0.80 (0.60–0.90)a	10.28 (8.00–13.00)a
P values	0.002	0.001	0.001	0.001

### 3.3. Apoptosis

The apoptosis score was significantly increased in OHSS group compared to the control group (P = 0.001), (Figure 2), (Table 2).

### 3.4. TRPM2 immunoreactivity

**Figure 3:**
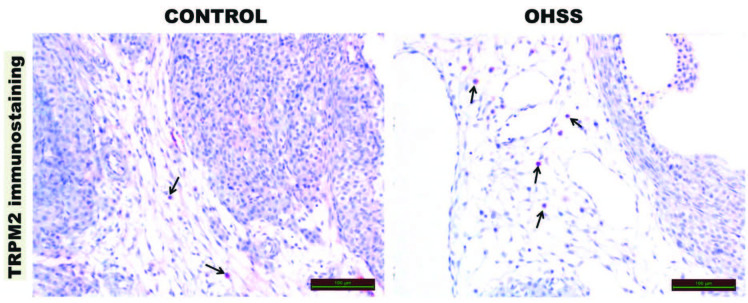
TRPM2 immune positive cells are observed in the ovarian stroma (black arrow).

TRPM2 immunoreactivity was significantly increased in OHSS group compared to the control group (P = 0.001), (Figure 3), (Table 2).

### 3.5. Biochemical findings

The levels of TNF‐α, VEGF and MDA were significantly increased in OHSS group compared to the control group (P = 0.007, P = 0.017 and P = 0.004, respectively) (Table 3). 

**Table 3:** TNF-α, VEGF and MDA levels of all groups.

Groups	TNF-α (ng/g protein)	VEGF (ng/mg protein)	MDA (nmol/mg protein)
G1	39.50 (23.37–56.26)	1.12 (0.85–1.42)	2.59 (1.73–4.35)
G2	61.84a (41.24–79.41)	1.75a (1.11–4.06)	4.67a (3.42–6.85)
P values	0.007	0.017	0.004

## 4. Discussion

In our study, we found a significant increase in TRPM2 immunoreactivity in OHSS group. Increased tissue MDA levels may be the result of impaired tissue oxygenation due to increased congestion or edema in hyperstimulated ovaries. In our study, TRPM2 immunoreactivity has been investigated for the first time in the pathophysiology of OHSS. Therefore, we think that our study may be a role model for future studies by pointing out this gap in the literature.

It has been shown that severe OHSS is associated with massively enlarged ovaries [22]. VEGF is a powerful vascular permeability mediator. It also plays a role in vascularization of the corpus luteum [23,24]. Pala et al. [25] have suggested that the relative hypoxia in enlarged ovaries in the rat model with OHSS may also contribute to the increase in VEGF. In our study, due to increased ovarian weight of OHSS group, we considered ovaries with OHSS as tumor tissues as every ovarian follicle has a rich microvascular network [26]. Even though the corpus luteum is a temporary tissue, endothelial cells form one of the highest vascularized tissues in the body, representing more than 50% of all cells [27]. In our study, we detected significantly increased angiogenesis and ovarian weight in OHSS group.

Endothelial TRP channels have been shown to be associated with angiogenesis and vascular remodeling because they can activate endothelial cell proliferation, migration, adhesion, tubulogenesis, and intracellular signaling pathways associated with permeability [28]. Endothelial TRP channels can control angiogenesis by mediating Ca2+ input in response to extracellular growth factors such as VEGF and bFGF that are free in peripheral circulation with ischemic damage or growing tumors [29]. Alternatively, TRP channels perceive cellular stress factors such as an increase in ROS production or a decrease in Mg2+ levels, which ultimately stimulates arteriogenesis [30]. An increased volume of ovaries with OHSS will require an increase in angiogenesis as well as VEGF expression, as in tumor tissue. Increased TRPM2 activity in our OHSS group may also induce angiogenesis along with increased VEGF.

Reactive oxygen species (ROS) produced by inflammation and injured tissues cause disruption in the vascular barrier, causing edema formation as a result of increased endothelial permeability [31]. TRPM2 has been shown to mediate endothelial hyperpermeability induced by H2O2 in human pulmonary artery endothelial cells [32]. Suppression of TRPM2 expression has been shown to reduce renal fibrosis induced by unilateral urethral obstruction, and suppress inflammatory cell infiltration and release of pro‐inflammatory factors [33]. It is also found that TRPM2 suppression significantly reduces TNF‐α and IL‐1β expression induced by TGF‐21 in HK2 cells [33]. The increase in MDA levels, a marker of ROS level [34], together with VEGF and TNF‐α levels in our OHSS group may be due to ovarian hypoxia. TNF‐α is cytotoxic for the endothelial cells produced by corpus luteum [35]. According to our experiment, increased MDA levels in our OHSS group indicate an increase in ROS production and a hypoxic environment due to increased congestion and edema. Increased TNF-α level also suggests that OHSS causes an inflammatory response in the ovary tissue. We also found an increase in TRPM2 immunoreactivity scores with increased MDA and TNF-α levels and VEGF in our OHSS group. This may indicate to us that there may be a relationship between increased angiogenesis and vascular permeability and TRPM2 immunoreactivity in OHSS’ pathophysiology. We detected significant congestion and edema in our OHSS group. This shows the increase in permeability in the ovary formed OHSS. According to the results of our histopathological and biochemical parameters in our experiment, it is clear that we do not have enough evidence to say that TRPM2 plays a direct role in the pathophysiology of OHSS. 

Apoptosis of luteal endothelial cells in response to TNF‐α and shrunken nuclei, DNA fragmentation and caspase-3 activation are all associated with morphological and biochemical properties of apoptosis [35]. The high levels of TNF‐α in OHSS group showed that OHSS can cause endothelial damage in ovarian follicles. Increased TNF‐α levels may explain the significant increase in apoptosis score in OHSS group.

In our study, increased apoptosis in correlation with the increase in TRPM2 may suggest that OHSS may cause a TRPM2‐mediated injury in the ovarian tissue. TRPM2 activation has been shown to play a role in stimulating the apoptotic pathway [36]. Eraslan et al. [37] have suggested that TRPM2 inhibition is effective in preventing ischemic acute kidney injury by alleviating oxidative stress, inflammation and apoptosis. However, even though some studies support the paradigm which suggest that TRPM2 activation causes cell death through a continuous increase in intracellular calcium levels [9,38] or increased cytokine production that increases tissue damage [39]; a number of physiological model systems have suggested that calcium intake through TRPM2 channels is a protective step rather than being harmful. TRPM2 inhibition has increased cell damage in pyramidal neurons exposed to oxidative injury [40]. Based on the results, TRPM2 channels sensitive to oxidative stress can facilitate the formation of oxidative damage and apoptosis, as well as induce angiogenesis and have a protective effect on ovarian tissue from oxidative damage in OHSS. As TRPM2 activity increases the severity of OHSS, it may also be involved in limiting the severity of OHSS by taking part in ovarian homeostasis. This issue can be clarified by further physiological and biochemical experimental studies in which mild, moderate, and severe OHSS groups are included. 

Our study has some limitations: in order to prevent violation of animal rights, the study was performed in a small group of rats. As this was an experimental study, the results obtained from this study cannot mimic the results in humans. On the other hand, our study has some strong aspects. This was the first study that investigated the TRPM2 activity in OHSS and its pathophysiology from a different perspective and its results can pave the way for new studies. 

In conclusion, we showed an increase in TRPM2 activity along with increased tissue MDA levels, vascular congestion, apoptosis scores in OHSS-developed ovaries. The increase in TRPM2 channel activity gives a different point of view on OHSS physiopathology.

## Acknowledgments

This experimental study was financed by Fırat University, Scientific Research Grant (Project number: 15.02.2019-TF.19.16).

This experimental study is derived from the master thesis of Dr. Cengiz Şanlı.
